# Keeping Hospital Records

**Published:** 1918-09

**Authors:** 


### Keeping Hospital Records.

The business of handling this war is being looked after in minutest detail. An immense force is required to look after the business details of the hospital service, so that there may be nothing undone for the soldiers and nothing neglected that will relieve the anxiety of the families of those who are sick and wounded. Exact information concerning wounded and sick American soldiers admitted to hospitals over seas will be made immediately available to relatives or friends of the men under a plan being worked out at the war department.

Secretary Baker visited the office of Surgeon General Gorgas to look into the daily reports from the hospitals with a view to having them carded, catalogued and tabulated so that the most instant information can be given to all inquirers.

The hospital records, Mr. Baker said, will be brought here weekly by courier from France and thus it will be possible to give the exact nature of the wound or the disease from which the men are suffering.

The information will be available through the adjutant general.

The task of installing the system will be a big one, but the war secretary believes the information should be available, for in thousands of cases it will relieve unnecessary distress and doubt which follows appearence of the names of men on casualty lists as wounded, degree undetermined, or severely.

